# Multiple Pathways of Genome Plasticity Leading to Development of Antibiotic Resistance

**DOI:** 10.3390/antibiotics2020288

**Published:** 2013-05-30

**Authors:** Zeynep Baharoglu, Geneviève Garriss, Didier Mazel

**Affiliations:** 1 Département Génomes et Génétique, Institut Pasteur, Unité Plasticité du Génome Bactérien, Paris 75015, France; E-Mails: baharogl@pasteur.fr (Z.B.); ggarriss@pasteur.fr (G.G.); 2 Centre National de la Recherche Scientifique, Unité Mixte de Recherche 3525, Paris 75015, France

**Keywords:** integron, SOS, sub-MIC, reactive oxygen species, horizontal gene transfer, RpoS, CRP

## Abstract

The emergence of multi-resistant bacterial strains is a major source of concern and has been correlated with the widespread use of antibiotics. The origins of resistance are intensively studied and many mechanisms involved in resistance have been identified, such as exogenous gene acquisition by horizontal gene transfer (HGT), mutations in the targeted functions, and more recently, antibiotic tolerance through persistence. In this review, we focus on factors leading to integron rearrangements and gene capture facilitating antibiotic resistance acquisition, maintenance and spread. The role of stress responses, such as the SOS response, is discussed.

## 1. Introduction

Since the discovery of penicillin by Alexander Fleming in 1928, antibiotics have been the major line of attack for combating infectious diseases. Extensive use of antibiotics, whether natural (isolated from bacteria or fungi) or synthetic, gives rise to development of antibiotic resistance, making it difficult to treat various infections, especially in hospitals where multi-resistant bacteria are involved in nosocomial infections.

The mechanisms underlying bacterial resistance to antimicrobial agents reside in the ability of bacteria to quickly modify their genomes. This plasticity is a consequence not only of spontaneous mutations or genome rearrangements that can occur during the bacterial life cycle, but also of exogenous gene acquisition through genetic exchange between bacteria and gene capture in integrons.

An integron is characterized by (i) an *intI* gene encoding a site-specific recombinase of the tyrosine recombinase family, (ii) an adjacent primary recombination site *attI* [1] and (iii) an array of gene cassettes, downstream of a constitutive promoter (Pc). A gene cassette is a promoterless open reading frame (ORF), flanked by site-specific recombination sequences (*attC*). The integrase IntI recognizes *attC* and *attI* recombination sites and respectively catalyzes excision (*attC* × *attC* recombination) and integration (*attI* × *attC* recombination) of the cassettes, generating combinations that allow the expression of genes that have been positioned close to Pc [2]. Other than integron rearrangements, IntI also allows the integration of exogenous circular promoterless gene cassettes carrying the *attC* recombination site [3]. The integrated gene cassettes can be expressed from the Pc promoter located upstream of the *attI* site in the integron platform [4].

In this review we focus on the factors that lead to integron rearrangements and gene capture and that enable antibiotic resistance acquisition, maintenance and spread.

## 2. Integrons Are Major Contributors to the Development and Dissemination of Multiple Resistances in Bacteria

Early on, the effective treatment of infectious diseases has been impeded by the development of antimicrobial resistance. Single drug resistant phenotypes were not entirely unforeseen, as demonstrated in early laboratory studies. In contrast, however, multi-drug resistance was not anticipated since the co-appearance of multiple mutations conferring such a phenotype was considered to be beyond the evolutionary potential of a given bacterial population. However, bacteria sometimes possess adaptive genetic resources—termed integrons—that can be used as a reservoir of silent genes mobilizable when needed. Integrons are natural gene expression systems that allow the integration of a gene cassette by site-specific recombination, transforming it into a functional gene [5].

### 2.1. Resistance Integrons

Multi-resistance integrons (RI) have been isolated on mobile elements responsible for the assembly and rapid propagation of multiple antibiotic resistances in Gram-negative bacteria and are all embedded inside transposons [6,7]. Integrons were only formally identified as agents of multiple antibiotic resistance gene recruitment in the late 1980s [8]. However, they have contributed to the initial multidrug resistance outbreaks in the 1950s, as indicated by the involvement of Tn*21*—an integron-containing transposon—in the resistances disseminated by plasmid NR1 (R100) [9]. Several genera including *Enterobacteriaceae*, *Pseudomonas*, *Vibrio* and *Acinetobacter* (see for example [10,11]) have been shown to harbor RIs. To date, more than 130 different resistance cassettes have been identified in these elements, allowing their hosts to resist to all classes of antibiotics except tetracycline [12,13,14].

### 2.2. Chromosomal Integrons

Several integron classes have been described since 1998, and are in many cases sedentary components of the genome of a number of environmental bacteria, especially members of the proteobacteria group [15,16,17,18,19]. Among these chromosomal integrons, the ones found in all *Vibrio* species, in many *Pseudomonas, Treponema* [20] and in *Xanthomonas* harbor large arrays of gene cassettes (up to 200), in structures called superintegrons (SI) [5]. The SIs are defined by the fact that they show a large array of cassettes (>20 and up to 200) that all share a high degree of identity of their associated *attC* recombination sites (>80%).

Evidence suggests that the SIs and the other chromosomal integrons are the source of the RIs and their cassettes. Phylogenetic relationships point to freshwater b-proteobacteria as the recent ancestors of the most common RIs (Class 1) [21]. Several SIs have been found to carry proto-resistance genes that can provide a resistance phenotype once recruited in a RI [13] (for a review, [14]). Among these are the *dfr* trimethoprim resistance cassettes [22], the *catB9* chloramphenicol resistance cassette [13], the ampicillin resistance cassettes *bla*^carb7^ and *bla*^carb9^ [23,24] and a more recently discovered *qnrVC* cassette conferring resistance to quinolones [25]. The discovery of integrons, and among these the SIs, in the chromosome of environmental strains has led to the extension of their role from the “simple” acquisition of resistance genes to a wider role in the adaptation of bacteria to different environments. Notably, all strains of *V. cholerae* carry a SI in their chromosome (for a review [5]).

Studies on the dynamics of cassette recombination and on the regulation of integrase expression are ongoing. Integrons carrying multiple resistance cassettes are stable in the laboratory, even in absence of selection, suggesting the integrase is expressed at low levels in these conditions. The cassettes are nevertheless transferred at a high rate in the environment [26], pointing to a high level of integrase expression in some conditions. Elucidating the mechanisms that regulate the expression of the integrase in various environments would shed light onto the conditions that govern the acquisition of new genes. In the case of resistance and adaptation genes ensuring better fitness in a given environment, this could facilitate the development of new strategies preventing it.

## 3. Connections between Integron Rearrangements and Horizontal Gene Transfer

### 3.1. Integron Integrase Expression Is Regulated by the SOS Response

**Figure 1:**
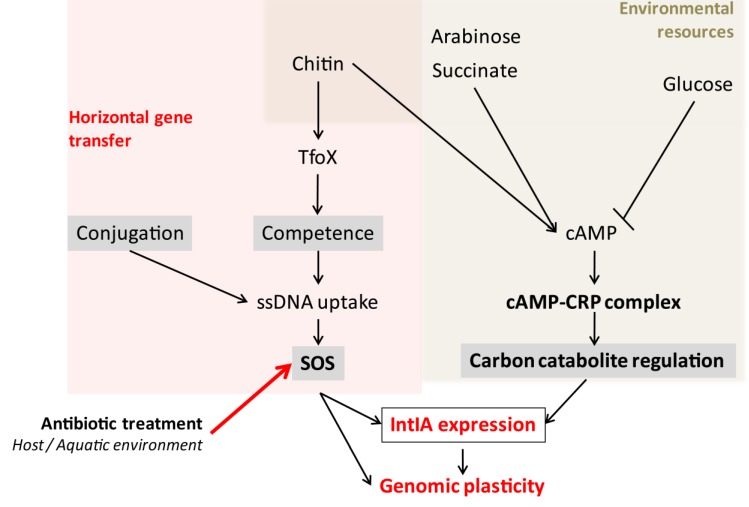
Model of *intIA* regulation and its implications for genome plasticity. Grey boxes represent mechanisms involved in IntIA regulation. Horizontal gene transfer (conjugation, transformation) induces SOS through ssDNA uptake by recipient cells, which in turn triggers *intIA* transcription. Carbon sources present in the environment also regulate IntIA expression through carbon catabolite control (adapted from [31], Copyright© American Society for Microbiology).

The SOS response is a bacterial stress response induced when an abnormal amount of single-stranded DNA (ssDNA) is present in the cell [27]. ssDNA is the substrate for RecA polymerization and the formation of a ssDNA-RecA nucleofilament. This activated state of RecA catalyzes the auto-proteolysis of a repressor, LexA, which normally represses the SOS regulon. Sequence analysis of the promoter regions of *intI* genes from various RIs and SIs has led to the identification of a conserved region of 16 nucleotides localized 20 to 40 nucleotides upstream of the *intI* gene. This sequence is similar to the *Escherichia coli lexA* boxes, which are the target for binding and repression by LexA [28]. It was further demonstrated that *intI* is indeed regulated by the bacterial SOS response: the activity of IntI from RIs and the *V. cholerae* SI is induced after treatment with mitomycin C, a DNA damaging agent known for inducing SOS. Moreover, the regulation of the *intI* promoter by the SOS response was confirmed in strains carrying different *lexA* alleles (constitutive or defective SOS mutants). *In vitro* tests have shown that LexA is capable of binding to and repressing the *intI* promoter [29,30]. Determining the conditions that lead to SOS induction is thus crucial to understand when and where cassette recombination takes place and how the integrase is activated. Figure 1 summarizes the current knowledge on integrase activation in various conditions.

The accumulation of ssDNA that triggers induction of the SOS response normally happens when cells try to replicate their damaged DNA. ssDNA is also produced by exogenous DNA uptake machineries that are involved in horizontal gene transfer (HGT), such as conjugation, transformation and occasionally transduction. Whether through conjugation or transformation, it is now clear that the integron plays a major role in bacterial adaptation to the local environment, as shown for *V. cholerae*, where integron-mediated interspecies gene capture allows for efficient adaptation to local growth conditions in a given environment [32].

### 3.2. Conjugation Induces the SOS Response and Integrase Expression

Conjugative transfer of several broad and narrow host range plasmids induces the SOS response in recipient *E. coli* and *V. cholerae* cells [33]. It had been previously shown that during inter-species Hfr conjugation, SOS is induced in the host cell [34,35], suggesting that the low level of homology impairs recombination of the incoming plasmid DNA with the chromosome and thus dramatically enhances the induction of the SOS response. This also suggests that the levels of SOS induction may reflect the ability of RecA to find homologous DNA molecules and initiate strand exchange [36]. In the case of plasmid conjugation, there is little or no similarity with the bacterial chromosome, which might explain the very high SOS induction levels observed. Interestingly, the strongest SOS-inducing plasmids are the ones that cannot replicate in the recipient cell, suggesting that even when the acquired exogenous DNA cannot be maintained, alterations in gene expression in the recipient cell can still occur and possibly lead to antibiotic resistance development. This is particularly relevant in environments where bacterial communities are concentrated. It was also proposed that narrow host range plasmids could constitute a pool of mobilizable suicide vectors that facilitate dissemination of resistance cassettes among evolutionarily distinct bacterial species without the fitness cost associated with plasmid maintenance [37].

Triggering integron cassette recombination is one of the consequences of the induction of the SOS response by conjugative DNA transfer [33]. The expression of the RI integrase IntI1, and subsequent cassette recombination, was studied in *E. coli* during HGT [33]. Conjugative transfer of strong SOS-inducing plasmids increased expression of IntI1, leading to an enhanced SOS-dependent cassette excision rate, linking conjugation, site-specific recombination and genome remodelling. An important consequence of this is the emergence of antibiotic resistant clones, as demonstrated by the acquisition and expression of a chloramphenicol resistance cassette (*catB9*) in the SI of an originally sensitive *V. cholerae*, through conjugation-mediated SOS induction [33].

*V. cholerae* alternates between two life styles: growth in biofilms on crustacean shells and colonization of the intestinal tract during infection [38,39]. In both cases, it cohabits with a variety of other bacteria. Induction of the SOS response can thus result not only in chromosomal cassette rearrangements, but also in the capture resistance genes by RIs located on conjugative plasmids that might later transfer to new hosts [5,40,41]. Moreover, conjugation commonly takes place in environments where bacterial populations are concentrated, such as wastewater treatment plants, and where they harbor a large diversity of integrons on conjugative plasmids [42,43]. A remarkable example of gene exchange between bacteria was shown for the Enterobacteria in the mammalian gut when, upon infection with pathogenic *Salmonella*, HGT was boosted between the invading bacteria and the resident *E. coli* [44]. Under these conditions the frequency of transconjugant formation approached 100% and lead to the spread of plasmid-encoded fitness, virulence and antibiotic resistance factors. If conjugation induces SOS in the recipient cell, and if the conjugative plasmid (or the host cell) carries an integrase, it is thus easy to imagine how rearrangements and gene capture can lead to the development and dissemination of resistance determinants, as was shown for *V. cholerae* [13]. This also underlines the role of stress in genome plasticity.

### 3.3. Natural Transformation induces the SOS Response and Integrase Expression

Transformation is a second mechanism of HGT that relies on ssDNA uptake and processing [45]. Transformation occurs when a bacterial cell reaches a competent state rendering it capable of taking up DNA present in its environment and, in some cases, of integrating the acquired DNA in its genome by recombination [46]. It was observed in *Bacillus subtilis* that when lysogenic strains were rendered competent, the induction of a prophage led to the reduction in the frequency of transformation [47]. Prophage induction is in many cases SOS-dependent. It is likely that the reduced transformation efficiency of lysogenic cells compared to non-lysogenic cells is the result of induction of the SOS response by the ssDNA acquired during transformation. Several mechanisms, such as special growth conditions or stress, lead to the induction of competence for natural transformation [48]. Competence has been suggested to be a stress response that could substitute for the SOS response in some bacterial species that lack an SOS regulon but in which the DNA repair genes are part of the competence regulon (for a review see reference [49]).

Transformation has been widely studied in several γ-proteobacteria (for a review see reference [48]). *V. cholerae* is one of the naturally competent gram-negative bacteria [50,51]. It is well established that natural competence for transformation in *V. cholerae* is regulated by TfoX (also called Sxy in *Haemophilus influenzae)*. Competence is induced in *V. cholerae* by the presence of chitin [50], a component of the crustacean shells that are one of its natural growth substrates. In addition to genes required for its metabolization, the presence of chitin triggers the expression of TfoX [52,53] and of the entire competence regulon [54,55,56,57].

Previously performed microarray studies following growth on chitin did not reveal an increase in the expression of the SI integrase or of the SOS regulon [58]. However, these studies were performed with the non-transformable *V. cholerae* laboratory strain, which naturally carries an inactivated *hapR* allele and is as such deficient in competence induction [59]. HapR is the major regulator of quorum sensing and it was shown that the expression of HapR is required for the *V. cholerae* natural transformation [59]. Moreover, no exogenous DNA was added to the medium in these studies. Redfield and collaborators also performed microarray analysis on *H. influenzae* and did not see any up-regulation of SOS regulated genes by TfoX/Sxy [57]. Again, no DNA was added to the reaction mixtures. However, when a *V. cholerae* HapR^+^ strain was used induction of the SOS response, as well as expression from the wild type SI integrase promoter, was detected upon addition of linear DNA to competent cells [31]. Thus, like conjugation [33], transformation is a mechanism of HGT that induces the bacterial SOS response and therefore also induces expression of the *V. cholerae* integron integrase.

In another study, gene capture by *Acinetobacter baylyi* integrons during natural transformation with DNA from various integron-harboring bacterial species was assessed [60]. The absence of an SOS regulon in *A. baylyi*, and thus absence of the LexA repressor, leads to a constitutively active integrase promoter. Remarkably, the transient presence of foreign DNA in the cytoplasm of the recipient strain was shown to be sufficient for efficient interspecies cassette exchange. These results are reminiscent of the ones obtained with the abortive conjugation assays mentioned above [33]. Moreover, even when the integron integrase of the recipient strain was inactivated, an integrase gene encoded by the incoming donor DNA could efficiently replace the inactive copy in the host cell and allow full functionality of the chromosomal integron.

The acquisition of resistance genes under these conditions did not appear to have a fitness cost, highlighting the fact that gene capture is not only efficient but also that captured gene cassettes can be stably maintained in the chromosome without a negative effect on cell fitness. Interestingly, the SOS response also regulates some toxin-antitoxin (TA) systems [61]. The presence of 13 cassettes encoding TA systems in the *V. cholerae* SI might play a significant role in integron maintenance [62].

### 3.4. Integrating Conjugative Elements, Integron Cassette Rearrangements and the SOS Response

RIs, like other mobile elements such as transposons and IS*CR2* elements are sometimes found associated with integrating conjugative elements (ICEs) of the SXT/R391 family [63,64,65]. ICEs are self-transmissible bacterial mobile elements that play a major role in the dissemination of antibiotic resistance genes in bacterial populations. They transfer by conjugation in a process similar to that of many conjugative plasmids, and their transfer was shown to induce the SOS response in recipients to the same extent to what was observed for conjugative plasmids [66]. However, their stable maintenance in their host cell usually depends on site-specific integration into the chromosome. A consequence of this feature is that they are also transmitted vertically with the host chromosome during cell division [67].

SXT/R931 ICEs constitute the largest family of ICEs studied to date and are predominant in *Vibrio cholerae*, in which integrons also play a major role in gene exchange and genome plasticity. ICEs of the SXT/R391 family are now known to be widespread in clinically and environmentally relevant species of *Vibrio* and related γ-proteobacteria and are commonly associated with the resistance to multiple antibiotics such as sulfamethoxazole (*sul2*), trimethoprim (*dfrA1*, *dfrA18*), aminoglycosides (*aphA*, *strBA*), chloramphenicol (*floR*) and in one case to cephalosporins (*bla*_CMY-2_) ([68] and references therein). The common set of conserved genes shared by all members of this family encodes the functions necessary for their integration/excision, conjugative transfer and regulation. In discrete regions of this conserved scaffold each ICE contains additional variable DNA sequences which, besides resistance to multiple antibiotics, can encode heavy metal resistance, toxin/antitoxin systems [69,70], c-di-GMP turnover proteins [71] and multiples genes of yet unknown function [64]. C-di-GMP is a bacterial second messenger molecule involved in the transition between the motile and biofilm lifestyles of *V. cholerae* and thus directly impacts its epidemic potential by contributing to its survival in the aquatic environment (biofilm formation [72]) and its ability to colonize the human intestine (motility [73]).

#### 3.4.1. ICE Transfer Induces Integron Integrase Expression

In manner reminiscent of the induction of the bacteriophage λ lytic cycle, the excision/integration and dissemination of SXT is triggered by exposure to DNA damaging agents. However, the expression of Int_SXT_—the ICE-encoded tyrosine recombinase required for both integration and excision of SXT from the chromosome—is not under the control of LexA, but rather under the control of two ICE-encoded transcriptional activators, SetC and SetD [74,75]. These transcriptional activators are themselves repressed by a master repressor, SetR, which bears similarities with the λ cI repressor. Repression by SetR is alleviated in conditions which induce the SOS response [74] and by analogy with λ cI it is thought that the intracellular pool of SetR is depleted by the action of RecA bound to ssDNA. Thus, exposure to antibiotics that induce the SOS response triggers the excision and transfer of SXT/R391 ICEs. Recently, it has been shown that most antibiotics induce the SOS response in *V. cholerae* [66], a phenomenon which would lead not only to the expression of a co-resident integron integrase, but also to increased conjugative transfer of SXT/R391 ICEs. Conversely, conjugative transfer of SXT from an *E. coli* strain to a *V. cholerae* strain has been shown to induce the SOS response and cause a 12-fold increase in the activity of the SI integrase (*intIA*) promoter [33]. Thus, transfer of SXT/R391 ICEs in *Vibrio* strains is expected to lead to cassette rearrangements in the co-resident SI.

SXT/R391 ICEs are even further linked with integrons since a number of these elements contain a class 4 RI conferring resistance to trimethoprim (*dfrA1*) embedded in their sequence [30,64,76]. In all instances, this integron contains the same array of cassettes, to the exception of the one found in ICE*Vch*Moz10, which lacks the *dfrA1* cassette [63,64]. The absence of this cassette is the only marked difference between ICE*Vch*Moz10, isolated in Mozambique in 2004 and ICE*Vch*Ban9, isolated in Bangladesh in 1994 [64]. It is tempting to speculate that this RI, which contains four other cassettes of unknown function, might confer an advantage beyond the resistance to trimethoprim that allowed its conservation across time and geographical distance. In contrast to the integrases of other RI classes, the integrase of the RI found in class 4 RIs, IntI9, is not predicted to be induced by LexA as its promoter region lacks a LexA binding site, and the conditions which induce its expression remain to be determined [30].

#### 3.4.2. DNA Damaging Agents Increase ICE Plasticity

Besides leading to increased integron cassette rearrangements, induction of the SOS response has been recently shown to participate in ICE plasticity through the induction of an ICE-encoded homologous recombination system capable of promoting the formation of hybrid ICEs [77,78]. This recombination system (*bet*/*exo*) is related to the Red recombination system encoded by bacteriophage λ. It is under the control of the ICE-encoded transcriptional activators SetCD and thus induced by exposure to DNA damaging agents [78]. SXT/R391 ICEs all share the same chromosomal attachment site (*prfC*) and their ability to co-exist in the same cell allows for the integration in tandem of two similar but non-identical ICEs [79]. Inter-ICE recombination events occur between two tandemly arranged SXT and R391 ICEs and lead to the formation of novel ICEs composed of sequences derived from both parental elements present in the donor cell [77,79,80], thus potentially participating in the diversity of the antibiotic resistance patterns carried by these elements. The discovery that this homologous recombination system is induced during the SOS response sheds new light on the adaptation of these mobile genetic elements to their host’s stress response and to the extent with which they profit of this for their own plasticity. Exposure to DNA damaging agents such as antibiotics increase hybrid ICE formation and ICE conjugative transfer which, in *V. cholerae*, also contributes to inducing the SOS response. Ultimately this leads to increased integron integrase expression, ICE diversity and to the spread of antibiotic resistance determinants.

Interestingly, homologues of SXT *bet*/*exo* have been recently identified for the first time in a wide variety of bacterial species and on a number of conjugative elements, such as plasmids belonging to the A/C incompatibility group [64,78]. IncA/C plasmids are the closest parents of SXT/R391 ICEs and are largely recognized for their major role in multidrug resistance in *Salmonella*, *Yersinia pestis* and in aquatic γ-proteobacteria such as *V. cholerae* [81,82,83,84,85]. These broad host range plasmids also often carry class 1 RIs and encode, amongst others, resistance to β-lactams (*bla*_SHV-1_, *bla*_CMY-2-1_, *bla*_CMY-2-2_), tetracycline (*tetRA*), sulfonamides (*sul1* in the 3'-CS of the RI, *sul2*), aminoglycosides (*aadA* integron cassette, *aphA*, *strBA*), chloramphenicol (*cmlA7* integron cassette), and mercury (*merRTPCADE*) [64,85,86,87].

## 4. Integron Rearrangements and Cassette Expression are Triggered by Stress Caused by Carbon Source Limitation

Bacteria have the ability to adapt to various environments and efficiently switch between widely different growth conditions. For instance, *V. cholerae* alternates between two niches: the host’s intestines, which are full of nutrients, and the aquatic environment (crustacean shells) where nutrients are scarce and carbon sources vary.

### 4.1. Carbon Catabolite Control Regulates Natural Competence

When the level of the favorite carbon source (such as glucose) is low in the medium, bacterial cells start to use “slow” carbon sources (such as chitin in the case of *V. cholerae*), which activates the carbon catabolite control regulon, also called CRP (cAMP receptor protein) regulon. Carbon catabolite repression, defined as the inhibition of gene expression by the presence of the favorite (*i.e*., rapidly metabolizable) carbon source, has been widely studied [88,89,90]. In γ-proteobacteria, growth in media lacking the “favorite” carbon sources leads to increased expression of the CRP-regulated genes, through the activation of the adenylate cyclase [91]. Under such conditions, the rising levels of intracellular cAMP control the CRP regulon through the action of the CRP-cAMP complex (referred to as CRP in the following lines). CRP binds to regulatory DNA sequences termed *crp* boxes [92] and modulates transcription. CRP not only controls specific catabolic pathways involved in carbon metabolism, but also many other genes involved in important aspects of cell physiology and interaction with the environment [93,94], such as quorum sensing, virulence and competence [95,96,97]. Many genes involved in the adaptation and survival of *E. coli*, such as the *ccd* toxin, or the plasmid F *tra* genes, are also known to be regulated by CRP [98]. In *V*. *cholerae*, CRP represses the cholera toxin and the toxin co-regulated pilus [99], favoring growth in low-nutrient environments (outside the host) and virulence in nutrient-rich environments such as the intestine.

Interestingly, regulation of natural competence for transformation also depends on the CRP-regulon, mainly because an increase in cAMP levels triggers the expression of the competence regulon activator TfoX/Sxy [57,91]. Additionally, Δ*crp* mutants produce lower levels of HapR [100], essential for competence [50]. Finally, TfoX/Sxy, in complex with CRP, is involved in the induction of some competence-specific *crp* boxes [91,101,102]. *H. influenzae* and *V. cholerae* Δ*crp* mutants are thus unable to become competent [57,103]. Hence, carbon source-dependent regulation plays a role in genetic exchange through the regulation of natural competence for transformation.

### 4.2. Carbon Catabolite Control Regulates SI Integrase Expression

In *V. cholerae* CRP activation also acts by directly impacting the SI *intIA* integrase promoter activity in an SOS-independent manner [31]. The cAMP-CRP complex was in fact shown to directly bind the *intIA* promoter and activate its transcription, favoring cassette rearrangements in the SI [31]. CRP also enhances transcription from the primary cassette promoter Pc in the *attI* site (Krin and Mazel, unpublished results). Like the SOS response, CRP regulation can thus be considered a stress response because it modulates the expression of the catabolite control regulon when rapidly metabolizable carbon sources are scarce [88,89,90,94,104]. The regulation of the SI integrase by two different stress responses highlights the influence of the environment on bacterial genetic adaptability. One may speculate that outside the infected host, integron-harboring bacteria shuffle gene cassettes, creating genetic diversity in order to increase their odds of surviving in hostile conditions or when exposed to antibiotics.

## 5. Integron Rearrangements are Triggered by Stress Caused by Exposure to Sub-Minimal Inhibitory Antibiotic Concentrations

### 5.1. General Biological Effects of Sub-MICs of Antibiotics

A large proportion of the antibiotics ingested are released intact in the environment [105,106] and found at trace levels or as gradients in various environments [107]. This is particularly relevant in the aquatic environment [108,109] and in the mammalian hosts of pathogenic and commensal bacteria, where antibiotics can play a very important role in the selection of resistant bacteria [110]. These low concentrations do not affect bacterial growth and are referred to as sub-MICs, for sub-minimal inhibitory concentrations.

Unlike above-MICs, the biological effects of sub-MICs of antibiotics have not been studied in detail. Transcriptome and proteome analyses have shown that many antibiotics exhibit contrasting properties when tested at low and high concentrations [111]: sub-MICs modulate metabolism through altered transcription whereas higher concentrations of antibiotics inhibit growth (for a recent review, [112]). It has been noted for example that sub-MICs induce several changes in the expression profile of a wide range of genes unrelated to the target function, such as resistance to oxidative stress, motility, virulence and biofilm formation [113]. For instance, in *Salmonella enterica*, a sub-MIC of rifampicin was shown to modulate transcription of several promoters through the interaction with RNA polymerase [114], whereas sub-MICs of fluoroquinolones (FQs) modulate gene expression by inducing the SOS response through DNA damage [115]. Sub-MICs of macrolides also have an effect on gene expression in this bacterium [116]. It was thus proposed that antibiotic sub-MICs act as agents for bacterial communication and signaling. This would explain the natural occurrence of such concentrations in bacterial communities [113,117]. Biofilm formation (modified flagellar formation and motility) is another consequence of the exposure to sub-MICs of FQs, macrolides and vancomycin (*Staphylococcus sp.*) [118,119,120] and to sub-MICs of aminoglycosides (AGs) (*P. aeruginosa* and *E. coli*) [121]. Biofilm communities are known to be more resistant to antimicrobial agents [122]. Although the aim of this review is not to expose links between biofilms and resistance it is worth mentioning that biofilm-specific tolerance to FQs has recently been studied in detail [123] and that many reports describe SOS induction [124,125] and high horizontal gene transfer levels in biofilms [126,127].

### 5.2. Sub-MICs of DNA Damaging Antibiotics Induce the SOS Response

We depicted earlier in this review how SOS induction triggers integron rearrangements. Several classes of antibiotics (fluoroquinolones [128], β-lactams and trimethoprim [129,130,131]) are known to induce the SOS response and increase mutation frequencies in *E. coli*. By directly targeting DNA related functions (such as replication and repair) or the DNA molecule itself (through crosslinks or lesions) they lead to the accumulation of DNA damages. The resulting induction of the SOS response increases the frequency of mutations (shown for FQs and trimethoprim in *Staphylococcus aureus* [132,133]) and higher levels of homologous recombination (shown for FQ in *E. coli* [134]). Point mutations can result in antibiotic resistance acquisition. For instance, resistance to ciprofloxacin (FQ) and to rifampicin is due to mutations caused during the induction of the SOS response, through the action of the error-prone polymerases II/IV/V [135]. Sub-MICs of FQs were clearly shown to cause resistance development in *S. aureus* [136] and *S. enterica* [110]. The increased frequency of mutation in presence of FQs was proposed to lead to the overload of MutS-dependent mismatch repair, causing the accumulation of unrepaired DNA and the appearance of point mutations that lead to antibiotic resistance. On the other hand, antibiotics that do not target DNA—such as aminoglycosides (AGs), chloramphenicol, rifampicin and tetracycline—were initially discounted as SOS-inducers after studies in *E. coli* [66,131] and *S. aureus* [133]. Conversely, tetracycline was also shown to induce the appearance of mutations, which require SOS-regulated DNA polymerases [137], suggesting a link between tetracycline and the SOS response.

### 5.3. Sub-MICs of Non-DNA-Damaging Antibiotics Induce the SOS Response in Various Bacterial Species

Since many bacteria carry integrons that are under the control of the SOS response and that sub-MICs of antibiotics affect gene expression, it was essential to shed light onto the possible effects of sub-MICs on these genetic elements. Strikingly, and unlike for *E. coli*, sub-MICs of AGs, chloramphenicol, rifampicin and tetracycline induce the SOS response in *V. cholerae* [66], *Photorhabdus luminescens* and *Klebsiella pneumoniae* [138]. This unexpected induction of the SOS response suggests a role for intermediate factors that cause stress and lead to DNA damage in *V. cholerae*. Moreover, expression from the *V. cholerae* SI integrase promoter was shown to be induced in the presence of sub-MICs of antibiotics belonging to these classes [66]. A parallel can be made here with the effects of metronidazole, another antibiotic that does not cause direct DNA damage, on *Pseudomonas aeruginosa. P. aeruginosa* is known to be exposed to sub-MICs of antibiotics in the lungs of cystic fibrosis patients, where it causes chronic lung infections and where gradients of antibiotics exist. Strikingly, SOS-mediated integron rearrangements in *P. aeruginosa* led to β-lactam and ceftazidime resistance during metronidazole treatment of a patient [139]. It is interesting to note here that AGs and metronidazole are both capable of inducing the SOS response although neither causes direct DNA damage.

### 5.4. Induction of the SOS Response Promotes Acquisition, Maintenance and Spread of Antibiotic Resistances

#### 5.4.1. Acquisition of Antibiotic Resistance

Induction of the SOS response has other implications related to genome stability and antibiotic resistance [27,140]. On one hand, it facilitates acquisition of resistance. Induction of the SOS response increases the frequency of point mutations, as shown previously for FQs and ampicillin (mentioned above, [133]) and more recently for sub-MICs of AGs, rifampicin, tetracycline and chloramphenicol in *V. cholerae* [66]. Ampicillin at sub-MICs was found to down-regulate mismatch repair in *E. coli*, *P. aeruginosa* and *V. cholerae* hence increasing mutation frequencies [141]. The same phenomenon was observed in *V. cholerae* after treatment with AGs (Baharoglu and Mazel, unpublished results). Such modest increase in mutation frequency (from 10^−9^ to around 10^−8^) is of high importance since it was shown to influence the evolution of multidrug resistance in bacteria [142]. Indeed, strains characterized by low or high mutation rates actually have a lower resistance to antibiotics than strains that have an intermediate rate of mutation (around 10^−8^), and this, independently of the antibiotic tested [142]. Indeed, high mutation frequencies probably more often lead to deleterious mutations. Another way for FQ resistance acquisition through SOS induction is described in [143], where the *qnrB* gene conferring low resistance to quinolones was shown to be regulated by LexA. Resistance to quinolones is thus induced by the presence of quinolones themselves, at sub-inhibitory concentrations, or by other antibiotics that induce the SOS response. Another study has also suggested that increased resistance to one antibiotic (after sub-MIC FQ-dependent increase in mutation frequency) can lead to the development of resistance to other classes of antibiotics [133].

#### 5.4.2. Spread of Antibiotic Resistance

Moreover, induction of the SOS response can favor the spread of these resistances. As mentioned earlier, SOS induction leads to spread of antibiotic resistance genes by inducing the dissemination of integrating conjugative elements (ICEs) [74]. SOS induction can also facilitate HGT and dissemination of virulence factors carried by mobile genetic elements [144,145]. Interestingly, AGs, FQs and mitomycin C (MMC) induce the competence (*com*) regulon in *Streptococcus* [146]. *Streptococcus* does not have a homologue of the SOS repressor LexA; however, its *com* regulon is considered a parallel of the SOS regulon since it contains most of the DNA repair genes, including *recA*. This means that upon becoming competent for natural transformation *Streptococcus* also becomes highly recombinogenic, which favors the acquisition and expression of new genes.

#### 5.4.3. Conservation of Multiple Resistances

Apart from the evolution of bacterial resistance, sub-MICs are also involved in the conservation of multiple resistances by the bacteria carrying them, through reduced fitness cost. Selection and enrichment of resistant bacteria has been observed for *E. coli* and *Salmonella* using three different antibiotics (tetracycline, FQs, AGs) at a hundred-fold below the MIC [147]. The authors show in this study that in the presence of sub-MICs of these antibiotics, the fitness cost of antibiotic resistance is overcome and resistant bacteria are maintained. Similarly, bacteria that show slightly increased mutation frequencies and harbor antibiotic resistances are found in greater proportions in the commensal flora of cystic fibrosis patients subjected to prolonged antibiotic treatment [148]. Another study even concludes that resistant strains have a selective advantage over others in presence of sub-MICs of FQs and tetracycline [149].

### 5.5. Induction of the SOS Response Promotes the Development of Persister Cells

Finally, induction of the SOS response by antibiotics also leads to the formation of persister cells [150,151,152]. Persisters are antibiotic tolerant cells that are not killed during treatment and resume growth when antibiotics are removed (for a review [152]). Dorr *et al.* showed that persisters are not pre-existing dormant cells, but rather that their formation is induced by the SOS response [150]. Interestingly, the appearance of persister cells was shown to be much higher during treatment with a sub-MIC of FQ than when higher antibiotic concentrations were used (stronger SOS induction). Persister cell formation can occur through the induction of toxins from the toxin-antitoxin family, such as TisB from the SOS regulon, which decrease the growth rate (drop of ATP, no active peptidoglycan synthesis, no ribosome, no replication), causing tolerance to multiple antibiotics [151]. Interestingly, 15 toxin-antitoxin modules are present in the *V. cholerae* SI [76,153], and TisB may not be the only toxin that leads to persistence. Therefore, the sub-MIC use of SOS-inducing antibiotics in *V. cholerae* may lead to persistence and eventually contribute to the development of multidrug resistance.

### 5.6. Linking the SOS Response, Reactive Oxygen Species Formation and Oxidative Stress Response in the Presence of Sub-MICs of Antibiotics

The induction of the SOS response by sub-MICs of antibiotics that are not known to cause DNA damage formation is intriguing. A recent study demonstrated that β-lactams, FQs and AGs lead to cell death through the production of reactive oxygen species (ROS) in bacteria [154]. ROS damage DNA and damaged DNA is a potent inducer of the SOS response. This study suggests that all bactericidal antibiotics, regardless of their cellular target, have the potential to induce the bacterial stress response. Since ROS can damage DNA and proteins, and thus induce mutagenesis, [155,156] it could be the missing link between sub-MIC antibiotic treatment and the induction of the SOS response.

#### 5.6.1. Sub-MICs of Aminoglycosides Lead to ROS Formation in *V. Cholerae*

Our group very recently showed that sub-MICs of tobramycin leads to an increase of intracellular ROS formation in *V. cholerae*, causing oxidative stress even at concentrations 100 times below the MIC [138]. At sub-MICs, tobramycin mediates induction of the SOS response mostly through the formation of ROS and the subsequent 8-oxo-G incorporation in DNA. The effect on the SOS-dependent *intIA* promoter was also assessed and we demonstrated an increase in integron recombination in presence of of sub-MICs of antibiotics. This means that not only numerous rearrangements may take place within the *V. cholerae* SI, but that other SOS-regulated integrases [29,76] (such as those from plasmid-borne RIs) are likely to be induced if present in a *V. cholerae* cell. AGs are commonly used against Gram-negative bacteria and the fact that they induce integrase activation and gene capture is of particular concern. Induction of the SOS response by AGs is a conserved trait among distantly related Gram-negative pathogens such as *Klebsiella pneumoniae* and *Photorhabdus luminescens* [138]. *E. coli* on the other hand, has a stronger resistance to the stress triggered by AGs [138]. This might indicate that some species counter their poorly efficient protection system against oxidative stress by being more easily capable of modifying their gene expression patterns [157]. Interestingly, numerous cassettes encoding resistance to all AGs were characterized in RIs (to date, a total of 43 cassettes [14]). The capture and selection of these cassettes can now be explained by the fact that exposure to AGs directly induces the SOS response and thus, the integrase. This implies that the use of these antibiotics may promote cassette rearrangements and expression of integron-borne resistances to all families of antibiotics, including to ones that do not induce the SOS response in *E. coli.*

#### 5.6.2. Sub-MIC Driven ROS Formation Leads to Increased Resistance Development

Induction of ROS formation by sub-MICs of antibiotics was shown to increase resistance development in various bacteria. In *Proteus mirabilis* for instance, for which sub-MICs of FQs were also shown to stimulate ROS formation [158], repeated cultures in the presence of a sub-MIC of FQs induces the formation of FQ resistant variants. These resistant phenotypes were not due to the typical mutations in the primary targets of FQs (like the gyrase or topoisomerase IV) but rather to enhanced resistance to oxidative stress [158]. Additional data on sub-MICs of antibiotics underline their important consequence of allowing bacteria to survive to antibiotic concentrations that would normally be lethal. FQ treatment at high concentrations leads to lipid and protein oxidation by ROS. Strikingly, less oxidation was observed at high FQ concentrations when bacteria had first been exposed to sub-MICs of FQs [158]. In a sense, one can think of sub-MICs of antibiotics as “homeopathic doses” that protect bacteria when later exposed to a normally effective antibiotic treatment.

Interestingly, susceptibility to antibiotics increases in absence of ROS detoxification pathways [159,160]. In parallel, stabilization of a single oxidative stress-sensitive protein is sufficient to enhance oxidative stress resistance of *V. cholerae* [161], a fact that confirms the weight of protein oxidation on *V. cholerae*’s ability to cope with stress. Several very different modes of antioxidant molecule production have been discovered in bacteria and can have, for instance, a role in the stringent response [162] or in increasing superoxide dismutase and catalase levels through H_2_S production and therefore help counteract ROS formation during antibiotic stress [163]. By elevating the production of antioxidant enzymes, these mechanisms allow bacteria to grow in the presence of a wide range of antibiotics (ofloxacin, meropenem, colistin, gentamicin) and can be regarded as antibiotic tolerance mechanisms [164].

#### 5.6.3. Involvement of RpoS in the Response to Sub-MICs of Antibiotics

Oxidative stress is known to induce the RpoS regulon [165]. RpoS, the stationary phase sigma factor, is induced in response to various stresses during the exponential growth phase [166,167,168] and increases resistance to stress [169]. Genes expressed following the induction of the RpoS regulon, namely catalases (KatE, KatG) and iron chelators, protect cells from ROS-related DNA damage [170] such as double-strand DNA breaks [171,172,173]. RpoS was also shown to play a role in antibiotic tolerance: when screening for mutants with altered antibiotic tolerance and decreased persistence genes *dnaK*, *rssB*, *dksA* and *ygfA* were identified, among others [174]. The proteins encoded by these genes regulate the stability of RpoS and the expression of the RpoS regulon, which points out RpoS as one of the determinants of persistence development.

Furthermore, several lines of evidence suggest a role for RpoS in horizontal gene transfer. Recently, the RpoS pathway was shown to be linked with the expression of the integron integrase in *E. coli* in presence of sub-MICs of AGs [138]. Observations in *V. cholerae* also show that the integron integrase promoter is more active during the stationary growth phase, suggesting that there could be an effect of RpoS on the integrase promoter (Krin and Mazel, unpublished results). Furthermore, in *Pseudomonas knackmussii* RpoS controls the activation of the integrating conjugative element ICE*clc* [175]. In absence of RpoS, the ICE*clc* integrase promoter, which catalyzes ICE excision, is significantly less active. This considerably impairs horizontal transfer of ICE*clc*, as ICE excision is a prerequisite for its conjugative transfer.

RpoS is conserved within α-, β- and γ-proteobacteria, but the composition of the RpoS regulon varies from one species to the other [176]. It has been suggested that these variations sometimes arise from the integration of horizontally transferred genes into the RpoS regulon [176,177]. Environmental pressure can also cause rapid loss or change in the RpoS regulon [178,179]. An additional contribution to coupling stress and cassette array expression could reside in the integration of exogenous open reading frames, captured by the *V. cholerae* integron, into the RpoS regulon. Nonetheless, further work is still needed in order to characterize the possible effect of RpoS on RI and SI cassette array expression. RpoS has also been proposed to be involved in double-stranded plasmid transfer in *E. coli* in laboratory conditions [180]; however, the existence of such a mechanism in nature remains to be proven. Finally, quorum sensing enhances oxidative stress response and survival by up-regulating RpoS [181]. If one considers sub-MICs of antibiotics as signaling molecules that activate oxidative stress response, then RpoS can be named as one of the key players that trigger gene exchange and genome plasticity.

## 6. Conclusions

Sub-MICs of antibiotics appear to be potent agents of stress for bacteria. The SOS DNA damage response and the RpoS general stress response synergistically protect cells from this kind of aggressions. Horizontal gene transfer during conjugation and natural transformation also influences genome plasticity through acquisition of exogenous genes and through the induction of the SOS response. Moreover, several environmental factors such as carbon source or the presence of oxygen play a role in the activation of the integron integrase, as described throughout the manuscript. The possible effects of other factors such as pH, salinity and temperature are not excluded.

In the light of the studies mentioned in this review, we believe that it is important to better understand how different ecological niches and different lifestyles modulate the evolution of bacterial stress responses, since they have a major impact on the evolution of genome plasticity and antibiotic resistance. Although no data on the induction of the SOS response in the mammalian gut is currently available, several lines of evidence suggests this is a possibility: (i) the presence of sub-MICs of antibiotics in the gut, (ii) increased levels of HGT [44] and (iii) other factors like oxidative stress can plausibly be expected to induce the SOS response and, ultimately, integron rearrangements.

In the search for compounds that can potentiate the effect of antibiotics on bacteria, the ones that amplify ROS production are currently of high interest. Studies showed that bacterial metabolites can render *E. coli* persister cells more susceptible to AGs in the presence of certain carbon sources [182] and it was further proposed that the amplification of ROS production could have the same adjuvant effect on antibiotics [183,184]. These authors have identified 133 reactions that could be potential sources of ROS and demonstrated that the modification of these pathways can lead to increased antibiotic susceptibility through increased ROS formation.

An engineered bacteriophage that suppresses the SOS response (by over-expressing the LexA repressor) has also been reported to enhance the lethal effect of quinolones, AGs and β-lactams on *E. coli*, to reduce the number of resistant bacteria that arise from the antibiotic treatment, and to increase survival of infected mice [185]. According to the authors, these observations would be the result of disabling DNA damage repair. Another way of preventing treatment failure is by combating the SOS-induced mutagenic DNA polymerase-dependent mutations that lead to FQ resistance [135].

Unraveling the factors that control the expression of integron integrases is essential to determine the pertinence of the development of integrase inhibitors in the battle against the dissemination of multi-resistant strains. Understanding the molecular mechanisms that drive the emergence of drug resistance can facilitate the design of more effective treatments.
